# PLGA-Based Drug Delivery Systems for Remotely Triggered Cancer Therapeutic and Diagnostic Applications

**DOI:** 10.3389/fbioe.2020.00381

**Published:** 2020-05-05

**Authors:** Xue Shen, Tingting Li, Xiaoxue Xie, Yi Feng, Zhongyuan Chen, Hong Yang, Chunhui Wu, Shengqi Deng, Yiyao Liu

**Affiliations:** ^1^Sichuan Industrial Institute of Antibiotics, Chengdu University, Chengdu, China; ^2^School of Life Sciences and Technology, University of Electronic Science and Technology of China, Chengdu, China; ^3^Center for Information in Biology, University of Electronic Science and Technology of China, Chengdu, China; ^4^Hospital of Chengdu University of Traditional Chinese Medicine, Chengdu, China

**Keywords:** PLGA-based DDSs, targeting, remotely triggered cancer therapy, imaging, theranostics

## Abstract

Intelligent drug delivery systems based on nanotechnology have been widely developed and investigated in the field of nanomedicine since they were able to maximize the therapeutic efficacy and minimize the undesirable adverse effects. Among a variety of organic or inorganic nanomaterials available to fabricate drug delivery systems (DDSs) for cancer therapy and diagnosis, poly(D,L-lactic-co-glycolic acid) (PLGA) has been extensively employed due to its biocompatibility and biodegradability. In this paper, we review the recent status of research on the application of PLGA-based drug delivery systems (DDSs) in remotely triggered cancer therapy and the strategies for tumor imaging provided by PLGA-based DDSs. We firstly discuss the employment of PLGA-based DDSs for remotely triggered cancer therapy, including photo-triggered, ultrasound-triggered, magnetic field-triggered, and radiofrequency-triggered cancer therapy. Photo-triggered cancer therapy involves photodynamic therapy (PDT), photothermal therapy (PTT), and photo-triggered chemotherapeutics release. Ultrasound-triggered cancer therapy involves high intensity focused ultrasound (HIFU) treatment, ultrasound-triggered chemotherapeutics release, and ultrasound-enhanced efficiency of gene transfection. The strategies which endows PLGA-based DDSs with imaging properties and the PLGA-based cancer theranostics are further discussed. Additionally, we also discuss the targeting strategies which provide PLGA-based DDSs with passive, active or magnetic tumor-targeting abilities. Numerous studies cited in our review demonstrate the great potential of PLGA-based DDSs as effective theranostic agent for cancer therapy and diagnosis.

## Introduction

The utilization of nanotechnology in drug delivery has been extensively supposed to alter the pattern of the pharmaceutical industries for the predictable future (Farokhzad and Langer, 2009; Lim et al., 2013; Farooq et al., 2019). Intravenous chemotherapy is one of the common cancer treatments. However, the normal tissues and organs will be damaged owing to the undesirable side effects caused by non-specific distribution of chemotherapeutic drugs and lack of specificity for tumor cell recognition (Jia et al., 2012; Shen et al., 2017). Besides, the repeated treatment with one single drug can cause multidrug resistance in tumor cells, compromising the anticancer effects of chemotherapeutic drugs (Yang et al., 2018). Currently, the rise of nanotechnology have provided an versatile drug delivery systems (DDSs) for the efficient treatment of cancer, which offers a solution to the problem of body tissue damage caused by non-specific distribution of traditional chemotherapeutic drugs (Shi et al., 2010). The developed DDSs with a diameter between 10 and 200 nm exhibits favorable pharmacokinetic property, prolonged systemic circulation time, sustained drug release profile, and enhanced intratumoral accumulation when compared to the free drugs (Hu and Zhang, 2012). With the continuous improvement of the performance of intelligent DDSs, it is possible to achieve (1) improved stability of hydrophobic drugs and the possibility of hydrophobic drugs for systemic administration; (2) targeted drug delivery; (3) combined delivery of two or more drugs or other therapeutic agents for combination therapy; (4) combined delivery of the therapeutic agents and imaging agents for the visualization of drug delivery; (5) favorable biodistribution and pharmacokinetic property of drugs and finally realizing the enhanced therapeutic effects and reduced side effects (Farokhzad and Langer, 2009).

Since liposomes were proposed as protein and drug delivery vehicles for the treatment of diseases in the 1960s, nanotechnology has had a huge impact on the development of novel DDSs for controlled drug delivery and combination therapy (Shi et al., 2010). A variety of inorganic or organic materials have been utilized to prepare novel DDSs including inorganic nanoparticles (Li et al., 2016), liposomes (Zhao et al., 2015), polymer micelles (Shi et al., 2014), and polymer nanoparticles (Yang et al., 2016; Shen et al., 2019) for the effective cancer treatment. Among all these nanocarriers, one kind of polymer nanoparticles based on poly(D,L-lactic-co-glycolic acid) (PLGA) have attracted considerable attention due to their unique physical and chemical properties, such as tunable particle size, regular morphology, large surface area, favorable pharmacokinetic property, excellent biocompatibility, and biodegradability (Deng et al., 2014; Yang et al., 2015, 2018; Shen et al., 2017, 2019). Polymer nanoparticles are colloidal particles ranging in size from 10 to 1,000 nm, which can be fabricated by using synthetic polymers, such as poly(butylcyanoacrylates) (PBCA), poly(lactic acid) (PLA), poly(D,L-lactic-co-glycolic acid) (PLGA), or natural polymers, such as chitosan, gelatin, and albumin (Bamrungsap et al., 2012; Mc Carthy et al., 2015). A majority of polymer nanoparticles are biodegradable and biocompatible and their reactive functional groups allows easy conjugation with other ligands or polymers. PBCA nanoparticles are commonly used to deliver some conventional drugs such as chemotherapeutic drugs or nucleic acids such as plasmids (Alyautdin et al., 1995; Schneider et al., 2008). However, polycyanoacrylic acid and alcohol as the hydrolysis products of PBCA are cytotoxic, which limits the application of PBCA as a nanocarrier in the field of biomedicine (Mc Carthy et al., 2015). Contrarily, PLGA and PLA have been preferentially used for the preparation of nanocarriers since they can be degraded into lactic acid and glycolic acid, which are natural and non-toxic and able to be eventually degraded into water and carbon dioxide (Graf et al., 2012). Moreover, PLGA has been approved for the medical applications by the United States Food and Drug Administration (FDA) (Hamori et al., 2015). The various targeting moieties can be introduced to the surface of PLGA-based DDSs, providing them with tumor-targeting capability. And the appropriate outer surface engineering (such as PEGylation) of PLGA-based DDSs may prolong the blood circulation time of the DDSs (Graf et al., 2012). Chemotherapeutic drugs, photosensitizers, photothermal agents, therapeutic gene/siRNA, small-molecule inhibitors, and other therapeutic agents can be easily loaded in the PLGA-based DDSs for tumor treatment (Lee et al., 2012; Zhao et al., 2015; Shen et al., 2017, 2019). Furthermore, the imaging agents can also be integrated into PLGA-based DDSs to acquire the imaging property for tumor diagnosis (Chen et al., 2016). Co-loading the therapeutic agents and imaging agents into PLGA-based DDSs can make them the potential candidates for cancer therapeutics and diagnostics (known as cancer “theranostics”) (Jia et al., 2012).

Several previous literatures have summarized the application of DDSs based on PLGA in cancer therapy and imaging from the aspects of preparation methods of PLGA-based nano- and microparticles, uptake of PLGA particles into cancer cells, controlled drug release property, and combination treatments (Rezvantalab et al., 2018; Swider et al., 2018; Kim et al., 2019). In this review, we introduce the recent status of research on the application of PLGA-based DDSs in remotely triggered cancer therapy and the strategies for tumor imaging provided by PLGA-based DDSs. We firstly emphasize the external stimuli-triggered cancer therapy approach and then discuss the strategies for tumor imaging provided by PLGA-based DDSs, the cancer theranostics in biomedical applications as well as the targeting strategies which endow the PLGA-based DDSs with passive, active or magnetic tumor-targeting abilities are further discussed. We introduced various types of PLGA-based DDSs in this review, some of them are nano-scaled, while the others are micro-scaled. Hao et al. (2015) have synthesized the tumor-targeted and gold nanoshell-surrounded PLGA-based nanoparticles (denoted as ANG/GS/PLGA/DTX NPs) for cancer chemotherapy and PTT, which were spherical with a size of about 200 nm. The nano-sized particles can more easily accumulate in tumor regions owing to the leaky vasculature and poor lymphatic drainage and their smaller size than the cell gaps of vascular endothelial cells of tumors (Torchilin, 2011; Bamrungsap et al., 2012; Shen et al., 2019). Nanoparticles are appropriate for various administration routes including intravenous administration. ANG/GS/PLGA/DTX NPs with exposure to an 808 nm laser irradiation displayed considerable tumor inhibition efficiency after the intravenous administration. Their pharmacokinetic parameters were further analyzed. The AUC of ANG/GS/PLGA/DTX NPs was higher than that of DTX solution with a 1.42-fold increase, and the clearance of ANG/GS/PLGA/DTX NPs was much lower than that of DTX solution. These results indicated that the nano-sized ANG/GS/PLGA/DTX NPs exhibited the prolonged blood circulation time and could improve the bioavailability of free DTX. Fang et al. (2015) have designed and synthesized the magnetic responsive PLGA microspheres contained DOX (DOX-MMS) for combined chemotherapy and hyperthermia, of which the average size was measured to be 2.4 μm. Benefiting from their hollow structure and large size, the quantities of loaded DOX in DOX-MMS were 6.2%, and the encapsulation efficiency of DOX was up to 85%. The micro-sized DOX-MMS were intratumorally administrated, and showed an effective tumor inhibition effect in 4T1 tumor-bearing nude mice with exposure to alternating current magnetic field (ACMF). Niu et al. (2013) have designed and constructed the gas-filled multifunctional polymer microbubbles (MPMBs) to co-encapsulate iron oxide nanoparticles and DOX for tumor lymph node detection and therapy. Although these gas-filled microbubbles are not suitable for intravenous injection, the gas-filled microbubbles can be used as the drug/gene delivery systems and they have been proven to be capable of enhancing the ultrasound signals when used as the ultrasound agent (Hernot and Klibanov, 2008; Eisenbrey et al., 2010).

## PLGA-Based DDSs for Remotely Triggered Cancer Therapy

Cancer is one of the most fatal diseases and has long been a threat to human health. Their heterogeneity and complexity make tumors grow aggressively, leading to a significant increase in patient mortality (Das et al., 2009; Mohanty et al., 2011; Parhi et al., 2012). The nanocarriers based on PLGA has been demonstrated to be the most promising DDSs due to their unique physical and chemical properties, such as tunable particle size, favorable stability, excellent biocompatibility, and biodegradability (Deng et al., 2014; Yang et al., 2015, 2018; Shen et al., 2017, 2019). PLGA-based DDSs can load hydrophobic and hydrophilic chemotherapeutic drugs, photosensitizers, photothermal agents, therapeutic gene/siRNA, and other therapeutic agents and display the prolonged blood circulation time and tumor-targeting capability after the appropriate surface modification, resulting in the improved antitumor efficacy (Lee et al., 2012; Schleich et al., 2013; Topete et al., 2014; Zhao et al., 2015; Luo et al., 2018). Remotely triggered cancer therapy can allow for selective and precise eradication of the tumors and the controlled release of chemotherapeutics by putting the external stimuli (photo, ultrasound, magnetic field, and radiofrequency) on targeted regions (Rai et al., 2010; Shenoi et al., 2013). Moreover, remotely triggered cancer therapy can determine when the treatment starts and how long it lasts, allowing for precise treatment and reduced systemic toxicity. The external stimuli-triggered cancer therapy approach [photodynamic therapy (PDT), photothermal therapy (PTT), photo-triggered chemotherapeutics release, high intensity focused ultrasound (HIFU) treatment, ultrasound-triggered chemotherapeutics release, ultrasound-enhanced efficiency of gene transfection, magnetic field-triggered cancer therapy, and radiofrequency-triggered cancer therapy] provided by PLGA-based DDSs will be briefly summarized as follows. Representative applications of PLGA-based DDSs for remotely triggered cancer therapy are listed in Table 1.

**TABLE 1:** Summary of recent applications of PLGA-based DDSs for remotely triggered cancer therapy.

**Remote trigger**	**Therapy**	**Formulation**	**Therapeutic agent**	**Administration route**	**Applications**	**References**
Photo	PDT	Ce6/iron oxide co-loaded PLGA	Ce6	Intravenous administration	Human oral epidermoid cancer treatment (KB cells)	Lee et al., 2012
	PTT, Chemotherapy	DOXO-loaded BGNSH-HSA-ICG-FA	DOX, ICG, gold nanoshells	Intravenous administration	Cervical and breast cancer treatment (Hela cells, MDA-MB-231)	Topete et al., 2014
	Photo-triggered chemotherapeutics release, PTT	ANG/GS/PLGA/DTX	DTX, gold nanoshell	Intravenous administration	Glioblastoma treatment (U87MG cells)	Hao et al., 2015
Ultrasound	HIFU treatment	Fe_3_O_4_/PLGA	–	Percutaneous injection	Breast cancer treatment (VX2 squamous carcinoma cell line)	Sun et al., 2012
	Ultrasound-triggered chemotherapeutics release	MPMBs	DOX	Percutaneous injection	Tumor lymph node treatment (VX2 squamous carcinoma cell line)	Niu et al., 2013
	Ultrasound-enhanced efficiency of gene transfection	PLGA/PEI/DNA	pDNA	Intravenous administration	Human prostate cancer treatment (DU145 cells)	Chumakova et al., 2008
Magnetic field	Magnetic hyperthermia, chemotherapy	DOX-MMS	DOX, γ-Fe_2_O_3_ nanoparticles (IOs)	Intratumoral administration	Breast cancer treatment (4T1 cells)	Fang et al., 2015
Radiofrequency	RF ablation	DLM@PLGA	DL-menthol (DLM)	Intratumoral administration	Human cervical carcinoma treatment (Hela cells)	Zhang K. et al., 2016

### Photo-Triggered Cancer Therapy

PLGA-based DDSs have been developed for the photo-triggered therapeutic application including the PDT, PTT, and photo-triggered chemotherapeutics release by employing an external light as a trigger. It has been demonstrated that photo-triggered cancer therapy could selectively and precisely eradicate the solid tumors by putting the laser probe on targeted regions, realizing the satisfactory antitumor efficacy (Su et al., 2015; Yan et al., 2016b).

**FIGURE 1:**
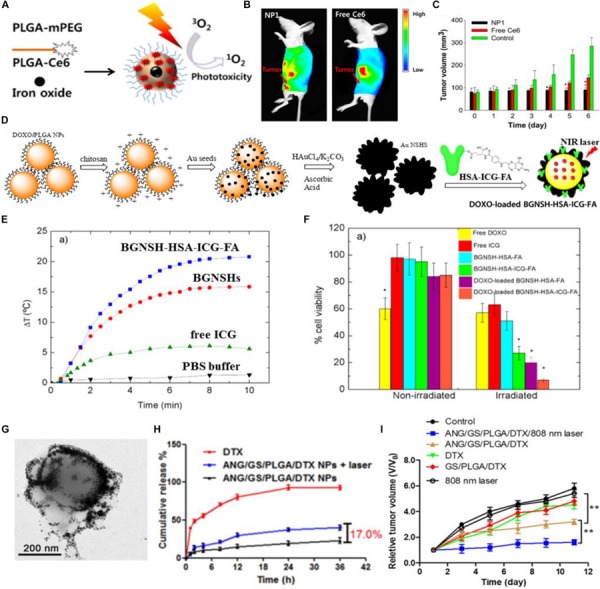
The photo-triggered cancer therapy based on PLGA-based DDSs. **(A)** Schematic illustration of Ce6/iron oxide co-delivered PEGylated PLGA nanoparticles (NP1). **(B)** The whole body fluorescence imaging of KB tumor-bearing nude mice after intravenous administration of Ce6/iron oxide co-loaded PEGylated PLGA nanoparticles (NP1) or free Ce6. **(C)** Tumor volume changes in KB tumor-bearing nude mice after intravenous administration of NP1 (equivalent Ce6 0.1 mg/kg body), free Ce6 (2.5 mg/kg) or PBS (control). Adapted with permission from Lee et al. (2012). Copyright 2012, Elsevier. **(D)** Schematic illustration of the synthetic process of DOXO-loaded BGNSH-HSA-ICG-FA. **(E)** The temperature profiles of BGNSH-HSA-ICG-FA, BGNSHs, free ICG and PBS buffer under continuous NIR laser irradiation (808 nm, 2 W/cm^2^). **(F)** Cell viability of the various nanoplatforms treated HeLa cells after 24 h of incubation in the presence and absence of NIR laser irradiation (808 nm, 2 W/cm^2^). Adapted with permission from Topete et al. (2014). Copyright 2014, American Chemical Society. **(G)** Transmission electron microscopy (TEM) image of ANG/GS/PLGA/DTX NPs after 808 nm laser irradiation for 5 min. **(H)** The *in vitro* drug release profiles of DTX and ANG/GS/PLGA/DTX NPs with or without laser irradiation treatment. **(I)** Relative tumor volume of various treatment groups. Adapted with permission from Hao et al. (2015). Copyright 2015, Elsevier.

Photodynamic therapy (PDT) can be used as a crucial approach for the treatment of various types of cancer by irradiating the photosensitizer-enriched tumor sites with light, the generating reactive oxygen species (ROS) can directly kill the cancer cells (Jang et al., 2011; Master et al., 2013; Chen et al., 2014). The photosensitizer-loaded PLGA-based DDSs have been widely concerned because of their good targeting capability and biocompatibility (Ricci-Junior and Marchetti, 2006). Importantly, the hydrophobic photosensitizers can be integrated into the PLGA-based DDSs, making it possible for the hydrophobic anticancer drugs to achieve the tumor-targeted delivery (Lee et al., 2012). Rojnik et al. (2012) designed the PEGylated PLGA nanoparticles for delivery of temoporfin, a effective second generation photosensitizer. In this study, the temoporfin delivered by PEGylated PLGA nanoparticles exhibited less dark cytotoxicity than the free temoporfin, while the phototoxicity of temoporfin-loaded PEGylated PLGA nanoparticles was not reduced when compared to the free temoporfin. The zinc(II) phthalocyanine (ZnPc)-loaded PLGA nanoparticles synthesized by Ricci-Junior and Marchetti (2006) showed the spherical morphology with a narrow size distribution and exhibited excellent biocompatibility due to their low dark toxicity assessed by the MTT assay. The significant photocytotoxicity of ZnPc-loaded PLGA nanoparticles showed them the great potential as the photosensitizer-loaded nanocarriers for PDT. Lee et al. (2012) have fabricated the PEGylated PLGA nanoparticles to co-load chlorin e6 (Ce6, a photosensitizer that can generate ROS upon the laser irradiation) and iron oxide (*T*_2_ contrast agent) for tumor diagnosis and PDT. Firstly, Ce6 and mPEG were respectively coupled to the terminal hydroxyl group of PLGA, and then the obtained PLGA-mPEG and PLGA-Ce6 were used to synthesize the multifunctional PLGA nanoparticles through the double emulsion method, and the feeding ratio of PLGA-Ce6 and PLGA-mPEG was 75%: 25% (w/w) (Figure 1A). The whole body fluorescence imaging of KB tumor-bearing nude mice after intravenous administration of Ce6/iron oxide co-loaded PLGA nanoparticles (NP1) showed a remarkable fluorescence signal of Ce6 in the tumor region, while the mice after intravenous administration of free Ce6 exhibited a weak fluorescence signal of Ce6 in the tumor region, demonstrating the efficient tumor targeting capability of Ce6/iron oxide co-loaded PLGA nanoparticles (NP1) (Figure 1B). The subcutaneous tumor volume of NP1 (equivalent 0.1 mg Ce6/kg body) treated KB tumor-bearing nude mice was about 1.5 or 3 times smaller than those of free Ce6 (2.5 mg/kg) or PBS treated nude mice (Figure 1C). The low-dose administration of NP1 (equivalent 0.1 mg Ce6/kg body) displayed better tumor inhibition effect than high-dose administration of free Ce6 (2.5 mg/kg), suggesting that the Ce6-loaded PLGA nanoparticles showed enhanced PDT for tumor.

Photothermal therapy (PTT) has been proposed to be an attractive method for solid tumor elimination, which utilizes the light-absorbing agents to convert light energy into heat energy, thus the generated local hyperthermia can destroy the cancer cells irreversibly without causing damage to the healthy tissues (Dong et al., 2016; Yan et al., 2016a; Wang et al., 2017). Compared to the radiotherapy, chemotherapy and surgery, PTT has been supposed to be a less invasive, controllable and efficient cancer treatment approach (Shen et al., 2015). A large number of nanomaterials have been reported to act as the light-absorbing agents for PTT, such as gold nanomaterials (Wu et al., 2017), carbon nanotubes (Robinson et al., 2010), and graphene (Markovic et al., 2011), which have strong absorption in the near-infrared region. Indocyanine green (ICG), as an organic molecule, is another kind of near-infrared light-absorbing agent (Li et al., 2017). Notably, the near-infrared light with a wavelength range of 650–950 nm has low phototoxicity to skin and tissues due to the minimal light absorption of skin and tissues in near-infrared region (Yu et al., 2016). Topete et al. (2014) have designed and synthesized a multifunctional nanoplatform for tumor diagnosis and therapy. As shown in Figure 1D, the primarily synthesized DOXO-loaded PLGA nanoparticles were subsequently modified with the chitosan biopolymer, then the Au seeds were deposited onto the surface of chitosan-modified DOXO-loaded PLGA nanoparticles, next the DOXO-loaded branched gold nanoshells (BGNSHs) were obtained in the presence of HAuCl_4_/K_2_CO_3_ and ascorbic acid through a seeded-growth surfactant-less method, and finally the human serum albumin (HSA)-ICG-FA conjugated and DOXO-loaded branched gold nanoshells (DOXO-loaded BGNSH-HSA-ICG-FA) were obtained by adsorbing the prefabricated HSA-ICG-FA complex to the DOXO-loaded BGNSHs. The photothermal efficiency of the nanoplatforms were further evaluated. As shown in Figure 1E, the temperature of BGNSH-HSA-ICG-FA was rapidly increased and the ΔT of BGNSH-HSA-ICG-FA was ∼19°C after 5 min of irradiation (808 nm, 2 W/cm^2^), as compared to those of BGNSHs (ΔT = 15°C), free ICG(ΔT = 6°C), and buffer solutions (ΔT = 1°C). The enhanced photothermal efficiency of BGNSH-HSA-ICG-FA was mainly due to the strong absorption of gold nanoshells and ICG molecules in the NIR region, implying the great potential of BGNSH-HSA-ICG-FA for PTT of cancer. The cell viability of HeLa cells treated with BGNSH-HSA-ICG-FA in the presence of NIR laser irradiation was much lower than that of HeLa cells treated with BGNSH-HSA-ICG-FA in the absence of NIR laser irradiation, indicating the remarkable photocytotoxicity of BGNSH-HSA-ICG-FA as a consequence of the hyperthermia generated from gold nanoshells and ICG molecules. And the cell viability of HeLa cells treated with DOXO-loaded BGNSH-HSA-ICG-FA in the presence of NIR laser irradiation was the lowest among all groups, suggesting the significant phototoxicity of DOXO-BGNSH-HSA-ICG-FA and their latent capability for combined chemotherapy and PTT of cancer (Figure 1F).

The light as an external stimuli has also been used for on-demand drug release from the PLGA-based DDSs at the suitable position (e.g., tumor region). The DOX and ICG co-loaded PLGA-based nanoparticles (DINPs) fabricated by Zheng et al. (2013) exhibited the faster DOX release property and enhanced cellular uptake of DOX and ICG in MCF-7 and MCF-7/ADR cells under NIR laser irradiation. Hao et al. (2015) synthesized the docetaxel (DTX)-loaded PLGA@Au nanoparticles, and then the angiopep-2, one kind of brain tumor-targeted peptide, was conjugated onto the gold nanoshell of DTX-loaded PLGA@Au nanoparticles via Au-S bond to form the tumor-targeted and gold nanoshell-surrounded PLGA-based nanoparticles (denoted as ANG/GS/PLGA/DTX NPs) for cancer chemotherapy and PTT. ANG/GS/PLGA/DTX NPs showed the excellent photothermal response and their structure observed by TEM was collapsed, the core-shell structure of the nanoparticles was also destroyed due to the local hyperthermia (Figure 1G). The *in vitro* drug release profile demonstrated the photo-triggered chemotherapeutics release property of ANG/GS/PLGA/DTX NPs. As shown in Figure 1H, the drug release of ANG/GS/PLGA/DTX NPs treated with 808 nm laser irradiation was fast and their final cumulative chemotherapeutics release percentage has increased by ∼17.0% compared to that of ANG/GS/PLGA/DTX NPs without 808 nm laser irradiation. The *in vivo* anti-glioma efficiency of drug-loaded NPs was further assessed, as shown in Figure 1I, ANG/GS/PLGA/DTX NPs with exposure to an 808 nm laser irradiation displayed considerable tumor inhibition efficiency, when compared to the GS/PLGA/DTX NPs and ANG/GS/PLGA/DTX NPs without laser irradiation, implying the promising application of ANG/GS/PLGA/DTX NPs in glioma-targeted chemotherapy and PTT.

### Ultrasound-Triggered Cancer Therapy

Ultrasound has been widely used in medicine for multifarious diagnostic and therapeutic purposes due to their advantages of feasibility and non-invasiveness (Ferrara, 2008; Zhang X. et al., 2014). This section will focus on ultrasound-triggered cancer therapy based on PLGA nano-/micro-particles which involved HIFU treatment, ultrasound-triggered chemotherapeutics release, and ultrasound-enhanced efficiency of gene transfection.

**FIGURE 2:**
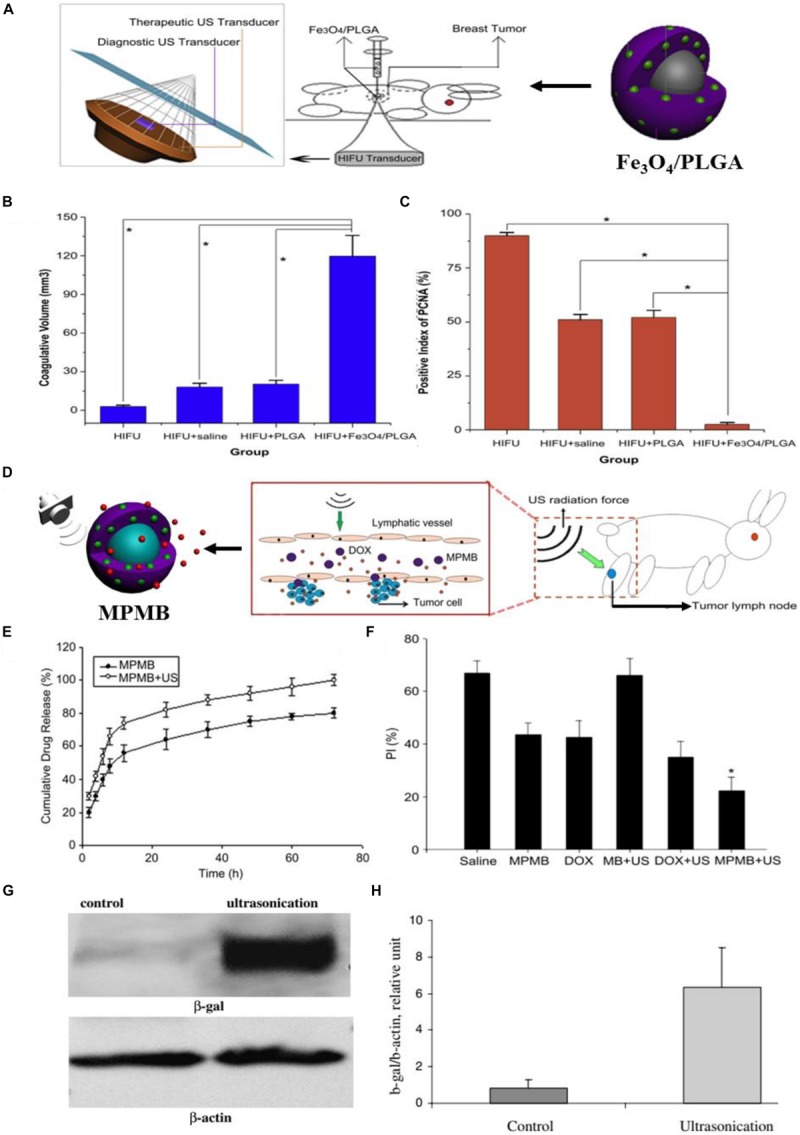
The ultrasound-triggered cancer therapy based on PLGA-based DDSs. **(A)** Schematic illustration of the developed Fe_3_O_4_/PLGA microcapsules used for HIFU ablation of breast cancer in rabbits. **(B)** The volume of coagulative necrosis in excised tumor tissues from different groups exposed to HIFU (*P < 0.05). **(C)** Positive index (PI) of PCNA in tumor tissues from different groups after HIFU ablation (*P < 0.05). Adapted with permission from Sun et al. (2012). Copyright 2012, Elsevier. **(D)** Schematic illustration of the process of MPMBs delivery into tumor lymph node and the controllable release of DOX from MPMBs triggered by low frequency US sonication. **(E)** The *in vitro* DOX release profiles of MPMBs with or without low intensity sonication. **(F)** The proliferative index (PI) of tumor lymph nodes in various groups. *P < 0.05 vs. the other groups. Adapted with permission from Niu et al. (2013). Copyright 2013, Elsevier. **(G)** The expression of β-galactosidase (β-gal) in control and ultrasound irradiated tumors assessed by western blot analysis. **(H)** Densitometry analysis of the β-gal expression from Figure 3G. Adapted with permission from Chumakova et al. (2008). Copyright 2008, Elsevier.

High intensity focused ultrasound (HIFU) is an advanced technology that was proposed for the first time in the 1940s (Manthe et al., 2010; Zhang K. et al., 2014). HIFU ablation has been demonstrated to be a feasible, non-invasive, and effective procedure for solid tumor treatment (Wu et al., 2004; Kennedy, 2005; Orsi et al., 2010). Sun et al. (2012) in their 2012 Biomaterials article, used PLGA to load the hydrophobic Fe_3_O_4_ nanoparticles for improving the therapeutic efficiency of HIFU ablation of breast cancer and realizing the ultrasound/magnetic resonance dual-modality imaging of tumors (Figure 2A). The obtained Fe_3_O_4_/PLGA microcapsules were characterized by uniform spherical morphology and an average diameter of 885.6 nm. The therapeutic effect of HIFU ablation for the breast cancer-bearing rabbits was further assessed. The tumor was treated with HIFU (150 W of acoustic power for 5 s) after percutaneous injection of Fe_3_O_4_/PLGA (saline and pure PLGA microcapsules was used as the control). After the treatment, the coagulative necrosis volume of the excised tumor tissues from the rabbits was calculated and the results showed a larger coagulative necrosis volume and lower positive index of proliferating cell nuclear antigen (PCNA) in tumor tissues of Fe_3_O_4_/PLGA microcapsules treated group when compared to the other groups treated with saline and the pure PLGA microcapsules (Figures 2B,C). These results were mainly attributed to the most obvious acoustic signal enhancement in the breast tumor region induced by the administration of Fe_3_O_4_/PLGA microcapsules and exposure to HIFU.

Ultrasound (US) can be used as another promising external trigger for on-demand drug release from the PLGA-based DDSs at the tumor region. Multifunctional polymer microbubbles (MPMBs) were designed by Niu et al. (2013) to co-encapsulate iron oxide nanoparticles and DOX for tumor lymph node detection and therapy. Figure 2D shows the process of MPMBs delivery into tumor lymph node and the controllable release of DOX from MPMBs triggered by low frequency US sonication. The workers recorded the *in vitro* DOX release profiles of MPMBs with or without low intensity sonication. As illustrated in Figure 2E, the cumulative drug release of MPMBs with low frequency US sonication showed an ∼90% of the released DOX from MPMBs, compared to that of MPMBs without low frequency US sonication showed <75% of the released DOX after 48 h. This result proved that the DOX-loaded MPMBs can be remotely triggered by US and then promote the DOX release from the microbubbles, showing the great potential for controllable drug release and accumulation of drugs into the targeted sites under US imaging guidance. The therapeutic efficacy of the MPMBs against tumor lymph nodes was further assessed. And the cell proliferation of the various treatment groups was evaluated in tumor lymph nodes model by using immunohistochemical staining methods. The results showed that the expression of PCNA could be observed in all groups and the proliferative index (PI) of tumor lymph nodes in the group treated with MPMBs and low frequency US sonication is substantially lower than those of any other groups (Figure 2F). However, no obvious difference for the PI was observed between the saline treated group and the pure MB (PLGA microbubble without loading iron oxide nanoparticles and DOX) combined with low frequency US sonication treated group. These results revealed that the developed MBMPs could remarkably improve the therapeutic efficacy against tumor lymph nodes when exposed to low frequency US sonication, which might be attributed to the controllable drug release behavior upon US irradiation and the cavitation effect.

Ultrasound has been utilized for gene delivery in recent years (Huber and Pfisterer, 2000; Pitt et al., 2004; Zarnitsyn and Prausnitz, 2004; Larina et al., 2005b). Larina et al. (2005a) has confirmed that combining the gene delivery systems based on nanoparticles with ultrasound may enhance the gene and drug delivery in targeted areas. Chumakova et al. (2008) fabricated the PLGA/PEI/DNA nanoparticles and combined them with ultrasound to improve the gene transfection efficiency in a nude mouse model. They firstly used PEI to absorb the β-galactosidase plasmids, and then the obtained PEI/DNA nanoparticles were loaded on the PLGA nanoparticles to form the PLGA/PEI/DNA nanoparticles. Polyethylenimine (PEI) as one type of cationic polymers has been used for gene delivery for the first time in the 1990s (Boussif et al., 1995). In recent years, PEI molecules with high molecular weight have been considered as one of the most efficient non-viral gene vectors for gene delivery (Gary et al., 2007). The PLGA/PEI/DNA nanoparticles were intravenously injected into the nude mice with DU145 human prostate tumors and then transfection efficiency was assessed by western blot analysis. As shown in Figures 2G,H, the expression of β-galactosidase showed an ∼8-fold enhancement in ultrasound irradiated tumors when compared to the control tumors. The enhanced transfection efficiency might be attributed to the changed structure of tumor cell membrane and tumor vasculature induced by ultrasound. These results indicated that the combination of PLGA/PEI/DNA nanoparticles with ultrasound was an efficient approach for *in vivo* gene transfection.

### Magnetic Field-Triggered Cancer Therapy

Magnetic hyperthermia induced by ACMF has been demonstrated to be a promising antitumor approach due to their relative non-invasive property (Jaber and Mohsen, 2013; Ramimoghadam et al., 2015). It has been proved that the magnetic nanoparticles (MNPs) could be used to produce magnetic hyperthermia via dipole relaxation under an external ACMF placed in the tumor regions, which can be employed to induce apoptosis of the tumor cells and make the tumor cells more sensitive to chemotherapy (Johannsen et al., 2007; Issels, 2008; Torres-Lugo and Rinaldi, 2013; Shen et al., 2015).

**FIGURE 3:**
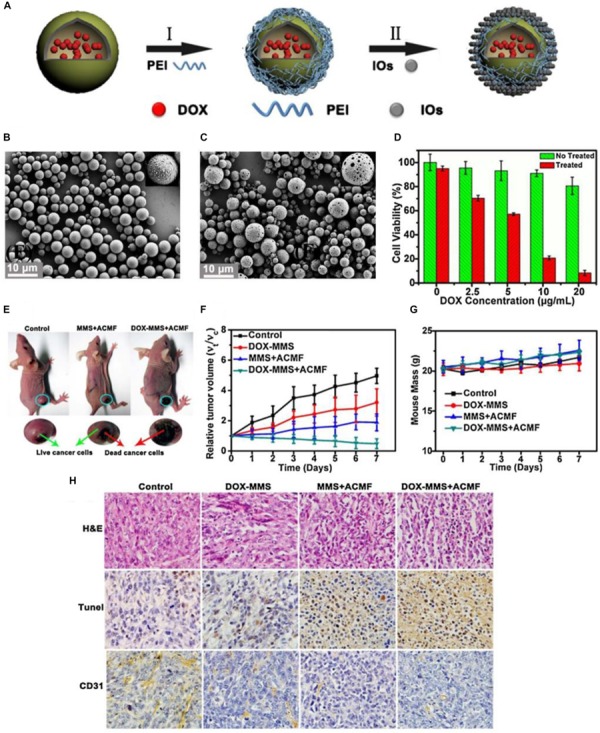
The magnetic field-triggered cancer therapy based on PLGA-based DDSs. **(A)** The preparation procedure of magnetic PLGA microspheres contained DOX (DOX-MMS). **(B)** SEM images of the DOX-MMS without exposure to ACMF. **(C)** SEM images of the DOX-MMS with exposure to ACMF. **(D)** Cell viability of the 4T1 cells treated with DOX-MMS at different DOX concentrations with or without exposure to ACMF. **(E)** The 4T1 tumor-bearing nude mice treated with PBS (control), MMS (containing no DOX) with ACMF, and DOX-MMS with ACMF after 7 days. **(F)** Relative tumor volume of various treatment groups. **(G)** Body weight changes in mice from the various treatment groups. **(H)** The hematoxylin and eosin (H&E) staining, TUNEL staining, and immunohistochemical anti-CD31 staining of the tumor tissues from mice in each group. Adapted with permission from Fang et al. (2015). Copyright 2015, Elsevier.

Fang et al. (2015) have designed and synthesized the magnetic responsive microspheres based on PLGA for combined chemotherapy and hyperthermia. Figure 3A shows the preparation procedure of magnetic PLGA microspheres contained DOX (DOX-MMS). Firstly, the workers in this study used the modified double emulsion solvent evaporation method to develop the DOX-loaded PLGA microspheres (DOX-MS). And then the positively charged polyethylenimine (PEI) was decorated on the surface of DOX-MS by electrostatic deposition to form an interlayer. Ultimately, the DOX-MMS were obtained by adsorbing the γ-Fe_2_O_3_ nanoparticles (IOs) on the surface of DOX-MS through the electrostatic incorporation. SEM images of the DOX-MMS after ACMF treated for 30 min showed that the DOX-MMS were broken and the pores could be observed on the surface of DOX-MMS (Figure 3C), while the SEM images of DOX-MMS without exposure to ACMF showed no obvious changes in morphology and almost all the microspheres could be observed to maintain the integrated structure (Figure 3B). The destroyed structure of DOX-MMS after exposure to ACMF was induced by the heat effect generating from the IOs on the PLGA shell triggered by ACMF, which can lead to an accelerated DOX release behavior of DOX-MMS. Figure 3D shows the cell viability of 4T1 cells treated with DOX-MMS at different DOX concentrations with or without exposure to ACMF for 30 min. There was an apparent DOX dose-dependent decrease of cell viability in the ACMF treated group. However, the cell viability of 4T1 cells without exposure to ACMF exhibited a slight decline with the increase of DOX concentration. The *in vivo* tumor inhibition assay revealed that the DOX-MMS treated with ACMF showed an effective tumor inhibition effect in 4T1 tumor-bearing nude mice models. The severe necrotic tumor tissues with dark gray color can be observed and the relative tumor volume in DOX-MMS treated mice with exposure to ACMF was the lowest among all experimental groups (Figures 3E,F). And there was no significant body weight loss observed in all groups (Figure 3G). The hematoxylin and eosin (H&E) staining, TUNEL staining, and immunohistochemical anti-CD31 staining of the tumor tissues from mice in all groups were further performed. As shown in Figure 3H, the visible necrotic areas, necrotic or apoptotic cells stained brown, and the low microvessel density of tumor tissues can be observed from the DOX-MMS treated mice with exposure to ACMF. These results indicated that the local hyperthermia triggered by the ACMF and the accelerated drug release behavior could enhance the antitumor effect of DOX-MMS.

### Radiofrequency-Triggered Cancer Therapy

Besides the ultrasound- and ACMF-triggered cancer therapy approach, the anti-cancer strategy triggered by radiofrequency (RF) has been extensively applied in various types of tumors (Miao et al., 2000; Dromain et al., 2002; Heynick et al., 2003). Although radiofrequency is a minimally invasive tool to inhibit tumor growth, the necessary high output power and long irradiation time of RF is needed, which can inevitably cause the damages to normal organs and tissues (Ryan et al., 2000). Magnetic metal nanoparticle have been validated to improve the ablated volume of tumors and in consequence promote the RF ablation of tumors, however, the high output power of RF is still needed to get high oscillating magnetic field (Kruse et al., 2011; Xu et al., 2012; Yun et al., 2014). Recently, the bubbles-induced cavitation has been proposed to be a promising strategy to enhance RF ablation of tumors.

**FIGURE 4:**
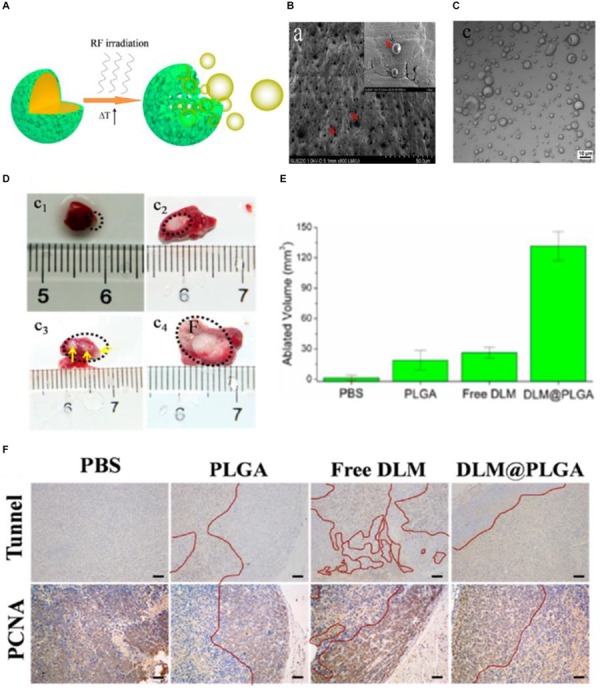
The radiofrequency-triggered cancer therapy based on PLGA-based DDSs. **(A)** Schematic illustration of an external RF-triggered RSV process mediated by DLM@PLGA. **(B)** SEM image of as-prepared DLM@PLGA (indicated by red arrows). **(C)** Confocal laser scanning microscope (CLSM) image of DLM@PLGA suspension after exposure to RF heating. **(D)** Optical images of the HeLa tumors treated with PBS (c_1_), PLGA (c_2_), free DLM (c_3_), and DLM@PLGA (c_4_) upon exposure to RF (1 W of the output power and 30 s of the irradiation time). **(E)** The corresponding ablated volumes of HeLa tumors calculated from Figure 5D. **(F)** Tunnel staining and PCNA immunochemistry analysis of tumor tissues, wherein the ablated and un-ablated regions were separated by the red curves (scale bar is 100 μm). Adapted with permission from Zhang K. et al. (2016). Copyright 2016, American Chemical Society.

Zhang K. et al. (2016) introduced the biocompatible DL-menthol (DLM) (melting point: 32–36°C) to a PLGA nanocapsule to construct a DLM encapsulated PLGA-based nanocapsule (abbreviated as DLM@PLGA). The solid DLM possesses the property of continuous solid-liquid-gas (SLG) triphase transformation, which is perfectly suitable for RF ablation (Zhang K. et al., 2014). The continuous cavitation triggered by radiofrequency solidoid vaporization (RSV) can result in the continuous enhancement of RF ablation in a reduced RF power output and shorten irradiation time manner. And the external RF-mediated local heat in the RSV process can trigger the vaporization of encapsulated solid DLM and resulting in DLM bubbles continuously generating from DLM@PLGA (Figure 4A). SEM image of as-prepared DLM@PLGA exhibited the uniform spherical morphology with an average particle size of 450 nm (Figure 4B). A plenty of DLM bubbles with different particle sizes can be observed in the confocal laser scanning microscope (CLSM) image of DLM@PLGA suspension after exposure to RF heating (60°C) (Figure 4C). The efficiency of RF ablation based on continuous cavitation was further evaluated in HeLa tumor-bearing nude mice models after intratumoral injection of PBS, PLGA, free DLM, and DLM@PLGA with exposure to RF (1 W of the output power and 30 s of the irradiation time). The obtained excised tumors from each group were used to calculate the ablated volume. As shown in Figures 4D,E, the DLM@PLGA treated HeLa tumors showed the largest ablated volume. And the three isolated ablation regions observed in the free DLM treated HeLa tumors were mainly resulting from the non-uniform distribution and agglomeration of free DLM induced by their hydrophobic property. Furthermore, tunnel staining and PCNA assay were further carried out to evaluate the molecular mechanism of the enhanced RF ablation. As shown in Figure 4F, the most apoptotic cells (brown color areas) but the least proliferating cells (brown color areas) were both observed in the DLM@PLGA treated HeLa tumors. These results demonstrated that the solid DLM encapsulated into the PLGA nanocapsules could strengthen RF ablation through the continuous cavitation which mediated by the RSV process triggered by RF field.

## Design of PLGA-Based DDSs With Imaging Property

In recent years, contrast agents have opened up a new development direction for tumor imaging, gradually become a new hotspot for cancer diagnosis and treatment (Chen et al., 2014). Applying the contrast agents into tumor diagnosis can realize imaging of tumors with a high spatial resolution and sensitivity (Shen et al., 2017; Yang et al., 2017). At present, the commonly used diagnostic methods in clinic mainly includes ultrasound imaging, X-ray computed tomography imaging (CT), magnetic resonance imaging (MRI), which can provide certain information for the preoperative staging and prognosis of tumors (James and Gambhir, 2012). However, because of some drawbacks of contrast agents including poor stability, rapid elimination from the body, and lack of targeting, the location of the lesions cannot be accurately located during diagnosis, which may lead to the compromised therapeutic effect of tumors (Kirchherr et al., 2009; Niu et al., 2013). It has been demonstrated that loading imaging agents into PLGA-based DDSs could improve the targeting ability and biocompatibility of imaging agents (Shen et al., 2017, 2019). The imaging agents can be encapsulated into PLGA-based DDSs or conjugated on to the outer surface of PLGA-based DDSs through the covalently linking with functional groups (such as carboxylic acid and hydroxyl groups) of PLGA (Martins et al., 2018). For example, the imaging agents which possess the amine groups can be covalently linked to the terminal carboxylic acid groups of PLGA by forming an amide linkage (Malinovskaya et al., 2017). Additionally, the imaging agents can also be introduced into PLGA by an appropriate linker such bifunctional PEG linker (El-Gogary et al., 2014; Park et al., 2014). *T*_2_-contrast agents based on superparamagnetic Fe_3_O_4_ nanoparticles can be encapsulated into the PLGA-based DDSs aiming for magnetic resonance (MR) imaging of tumors, which is a powerful and non-invasive imaging technique with relatively high temporal and spatial resolution (Li et al., 2013; Shen et al., 2017). A series of fluorescent dyes, near-infrared fluorescent imaging agents, and quantum dots, etc. can also be introduced into the PLGA-based DDSs aiming for fluorescence imaging of tumors, which plays an important role in the research of tumorigenesis and development due to its high sensitivity and low cost property (Edgington et al., 2009; Rotman et al., 2011). ICG, as a near-infrared (NIR) fluorescent imaging agent with low toxicity, has been used for cardiac function monitoring, liver output, and retinal angiography (Owens, 1996). Moreover, ICG can be utilized as a photoacoustic imaging contrast agent to produce the enhanced photoacoustic signal due to its strong NIR light absorbance, realizing the photoacoustic (PA) imaging of tumors in a non-invasive manner (Chen et al., 2016). It is noted that the gold nanomaterials could be encapsulated into the PLGA-based DDSs or coated onto the surface of the PLGA-based DDSs for PA imaging or X-ray CT (Hao et al., 2015; Song et al., 2015). The high-resolution tissue structure image of tumors will be obtained when employing the gold nanomaterials as the contrast agents for X-ray CT, which possesses the advantages of fast acquisition time, simple operation, and high availability (Li et al., 2010; Chen et al., 2014).

## PLGA-Based Cancer Theranostics

**FIGURE 5:**
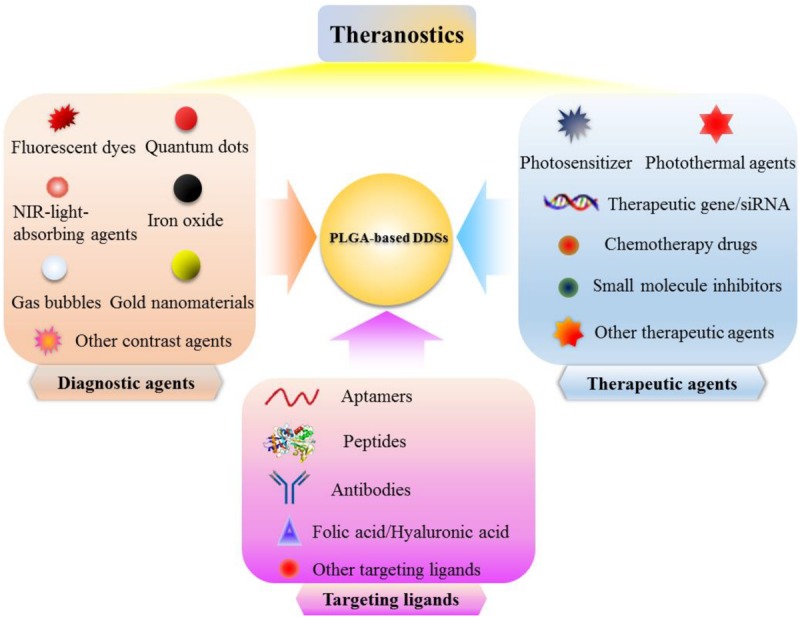
Schematic illustration of PLGA-based DDSs which integrated diagnostic agents, therapeutic agents, and targeting ligands for effective diagnosis and treatment of cancer.

Nowadays, clinical diagnosis and treatment of tumors are two separate processes, patients usually need to be diagnosed before treatment, and the two separate medical procedures are likely to delay the best time to treat diseases (Wang et al., 2012). With the rapid development of molecular imaging technology, various imaging modes have been studied to improve diagnostic imaging. Therefore, the development of a multifunctional nanoplatform with diagnostic and therapeutic functions has been the future development trend of nanomedicine (Song et al., 2017). By applying the nanoplatform which integrated diagnostic and therapeutic agents to tumor treatment, the entire process of chemotherapy can be monitored in real time, and whether the chemotherapeutic drugs are effectively delivered to the tumor sites can be monitored. The position, size change, and metastasis of the tumors can also be monitored to determine whether the chemotherapeutic drugs are effective in killing tumors. Many researchers have developed various types of PLGA-based DDSs that can be used for MRI, CT, fluorescence imaging, ultrasound imaging, and photoacoustic (PA) imaging of tumors to achieve the accurate tumor detection, meanwhile these PLGA-based DDSs can also be used to load the therapeutic drugs such as chemotherapeutic drugs, small molecule inhibitors, photosensitizers, photothermal agents and siRNA (Figure 5; Menon et al., 2013). Yang et al. (2018) have developed the charge-reversal PLGA-based ultrasound nanobubbles to co-load Dox and P-gp shRNA for reversal of drug resistance and enhancing the antitumor effect of chemotherapeutics. The *in vitro* and *in vivo* data substantiated that the drug and gene co-delivered PLGA-based nanobubbles could be used as an available theranostic agent for ultrasound imaging-guided chemotherapy and gene therapy of multiple drug resistance (MDR) tumors. Another novel PLGA nanoparticles coated with cancer cell membrane for dual-modal imaging-guided photothermal cancer therapy was prepared by Chen et al. (2016). The near-infrared light absorbing agent, ICG, was firstly encapsulated into the PLGA nanoparticles to obtain the ICG loaded PLGA nanoparticles which were employed as the core, then cancer cell membrane shell was coated onto the surface of ICG loaded PLGA cores by co-extruding the membrane vesicles and ICG loaded PLGA cores through a 220 nm polycarbonate membrane. The ultimately obtained cancer cell membrane-coated PLGA nanoparticles (ICNPs) with ICG loaded PLGA cores and cancer cell membrane shell were validated to have a favorable photothermal response and homologous targeting effect both at the cellular level and animal level. The results demonstrated that ICNPs could substantially accumulate into subcutaneous breast cancer tissues in MCF-7 tumor-bearing nude mice through homologous targeting and EPR effect, and can be used as the fluorescence imaging and photoacoustic (PA) imaging agents to clearly identify tumor locations and boundaries. The *in vivo* results also corroborated that ICNPs could eradicate the tumors upon exposure to NIR laser and prevent the recurrence of tumors, showing the great potential of the developed cancer cell membrane-coated PLGA nanoparticles as the versatile nanoplatform for homologous-targeting and fluorescence/photoacoustic imaging-guided PTT. Gu et al. (2016) have reported a facile approach to construct a multifunctional nanocapsule based on PLGA to co-load BSA capped gold nanoclusters (AuNCs) and ICG. Because of the carboxyl groups existed on the surface of AuNCs and ICG co-loaded mPEG-PLGA (AuIP) nanocapsules, the amino groups of RGD peptides can be coupled to AuIP nanocapsules by forming the amide linkage. The obtained AuIP-RGD nanocapsules were demonstrated to be able to specifically target the U87-MG cancer cells that overexpress integrin α_v_β_3_ by CLSM analysis. The results further validated the satisfactory performance of AuIP-RGD in both one-photon and two-photon fluorescence imaging of tumors as well as the PTT of tumors, showing the great potential of AuIP-RGD nanocapsules as the theranostic nanoplatform for tumor diagnosis and treatment applications.

## Targeting Strategies

The PLGA-based DDSs should be developed to achieve the effective drug delivery. In brief, the drugs loaded in the PLGA-based DDSs should be able to reach the targeted tumor site after administration, and the loss of drug activity and dose in the blood circulation before reaching the targeted site should be minimal. More importantly, the drug should only kill tumor cells without harmful effects on normal tissues and organs. Various modifications can be performed to fabricate the PLGA-based DDSs, such as changing their size, shape, structure, chemical and physical properties, etc. to make them accumulate into the tumor regions through passive targeting or active targeting approach (Torchilin, 2000; Farokhzad and Langer, 2009).

DDSs are inclined to passively extravasate through the leaky vasculature, which is the unique pathophysiological characteristics of solid tumors, and preferentially accumulate into tumor tissues through the passive targeting (Shen et al., 2019). Generally, the abundant tumor vasculature are leaky, and the pore size of the leaky vascular endothelial cell gap ranges from 100 to 800 nm (Torchilin, 2011). Meanwhile, the tumor tissues lack effective lymphatic drainage (Bamrungsap et al., 2012), which can lead to the decreasing diffusion process, ultimately resulting in the prolonged retention time of DDSs. Therefore, the DDSs with a particle size smaller than the pore diameter can easily penetrate the interstitium and finally become accumulated in tumor sites. The phenomenon of passive accumulation of DDSs into tumor tissues is referred to the enhanced permeability and retention (EPR) effect (Danhier et al., 2010; Greish, 2010).

**FIGURE 6:**
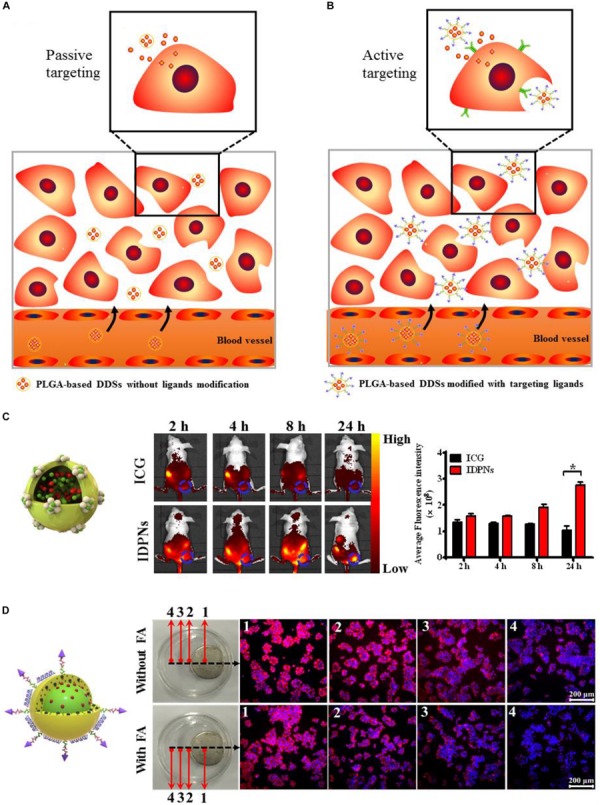
**(A)** Schematic illustration of the passive tumor-targeting approach of the non-ligand-modified PLGA-based drug delivery systems (DDSs). **(B)** Schematic illustration of the active tumor-targeting approach of the targeting ligand-modified PLGA-based DDSs. **(C)** The whole body fluorescence imaging of EMT-6 tumor-bearing nude mice after intravenous administration of free ICG and IDPNs and the fluorescent quantitative analysis of the tumor sites. Adapted with permission from Shen et al. (2019). Copyright 2019, American Chemical Society. **(D)** Fluorescence images of HeLa cells treated with LDM-PLGA/PPF nanocomposites for 8 h under exposure to an external magnet with or without free folic acid (FA) pretreatment. Adapted with permission from Shen et al. (2017). Copyright 2017, Dove Medical Press.

PLGA-based DDSs has been demonstrated to be the multifunctional DDSs that can target tumor sites owing to their characteristics such as small and tunable particle size, high stability, excellent biocompatibility, and simple surface modification (Deng et al., 2014; Yang et al., 2015, 2018; Shen et al., 2017, 2019). Appropriate surface modification (such as PEGylation, poloxamers and Tween 80 conjugation) of the PLGA-based DDSs can evade the phagocytic uptake by the reticuloendothelial system (RES) (Cai et al., 2016), leading to the prolonged circulation time in blood, which provide more opportunities for these surface-modified PLGA-based DDSs to accumulate into the tumor regions through passive targeting approach (Figure 6A). In our previously published literature, we have designed the PLGA-based nanoparticles modified with bovine serum albumin (BSA) to co-load near-infrared dye, indocyanine green (ICG) and chemotherapeutic drug, doxorubicin (DOX) for passive tumor-targeted combination cancer therapy (Shen et al., 2019). BSA modification can not only act as a surface stabilizer but also a biocompatible shell of the PLGA-based nanoparticles to evade the non-specific adsorption of plasma protein and the recognition of macrophage. The enhanced fluorescence signals of PLGA-based theranostic nanoplatform (denoted as IDPNs) were detected in tumor region after 24 h intravenous injection when compared to free ICG molecules, suggesting that the IDPNs possess the capability of passive accumulation into tumor sites via the EPR effect (Figure 6C).

To improve the targeting efficiency of PLGA-based DDSs to the targeted sites, affinity ligands such as antibodies (Venugopal et al., 2018), peptides (Graf et al., 2012), aptamers (Wu et al., 2010), or small molecules (Shen et al., 2012) can be introduced onto the surface of PLGA-based DDSs, which can be recognized by the specific receptors overexpresses on tumor cells and then bind tumor cells through ligand-receptor interactions, achieving the active targeting and accumulation of PLGA-based DDSs into tumor sites via the receptor-mediated cell uptake, as illustrated in Figure 6B. Folic acid (FA) is a kind of small molecule nutrient, as an non-immunogenic targeting ligand, it possesses high binding affinity to the folate receptor, which overexpresses on the surface of various types of human cancer cell membranes (Shen et al., 2012). Folate receptor (FR) has been extensively utilized for active tumor-targeting of nanocarriers via the receptor-mediated endocytosis (El-Gogary et al., 2014). Besides the passive and active targeting, magnetic targeting is another targeting strategy, which can be achieved by loading the magnetic nanoparticles into PLGA-based DDSs before exposing to an external magnetic field (Yu et al., 2016; Shen et al., 2017). Our group has developed the folic acid-conjugated PEGylated PLGA nanoparticles co-encapsulated with CdSe/ZnS quantum dots, doxorubicin and Fe_3_O_4_ nanoparticles followed by the adsorption of vascular endothelial growth factor (VEGF)-targeted small hairpin RNA (abbreviated as LDM-PLGA/PPF/VEGF shRNA) for tumor targeted drug delivery and cancer imaging (Shen et al., 2017). The folic acid modified on the surface of nanocomposites and the Fe_3_O_4_ nanoparticles encapsulated into the nanocomposites endow LDM-PLGA/PPF/VEGF shRNA with folate and magnetic dual targeting functions by folate receptor-mediated endocytosis and magnetic guidance. HeLa cells treated with LDM-PLGA/PPF nanocomposites exhibited the increase of intracellular DOX fluorescence signal along with the decreasing distance from the magnet, while an increased fluorescence signal of DOX in HeLa cells without free folate pretreatment was observed compared to the cells with free folate pretreatment, demonstrating the enhanced cellular uptake of LDM-PLGA/PPF by HeLa cells via magnetic guidance and folate receptor-mediated endocytosis (Figure 6D).

Currently, applying the intrinsic homologous adhesion property of the cancer cells to tumor targeting is another promising active tumor-targeting approach. Surface adhesion molecules such as galectin-3 or N-cadherin expressed on cancer cells have been proven to be the leading cause of multicellular aggregation formation. Therefore, the cancer cell membrane can be utilized for the surface functionalization of DDSs in order to obtain the homologous targeting capability. Chen et al. (2016) reported a cancer cell membrane-coated PLGA nanoparticles with encapsulation of indocyanine green (ICG) molecules for specific homologous tumor-targeted and photoacoustic/fluorescence imaging-guided photothermal cancer therapy. The results of the *in vivo* biodistribution and photoacoustic/fluorescence imaging showed a remarkably enhanced tumor accumulation of MCF-7 cell membrane-coated PLGA nanoparticles at 24 h post-injection when compared to the non-cancer cell membrane-coated PLGA nanoparticles in human breast cancer cells (MCF-7) bearing nude mice models. The illustrated examples validate that the PLGA-based DDSs with appropriate surface functionalization can provide a promising approach for passive tumor-targeting via the EPR effect or active tumor-targeting through ligand-receptor interactions, resulting in the enhanced delivery and accumulation of PLGA-based DDSs into tumors.

## Conclusion and Perspectives

In this review article, we summarize the recent status of research on the application of PLGA-based DDSs in remotely triggered cancer therapy and the strategies for tumor imaging provided by PLGA-based DDSs, specifically focusing on employment of PLGA-based drug DDSs for external stimuli-triggered cancer therapy including photo-triggered, ultrasound-triggered, magnetic field-triggered, and radiofrequency-triggered cancer therapy. These drug delivery systems based on PLGA were shown to possess excellent biocompatibility and biodegradability, uniform particle size, on-demand drug release behavior and external stimuli-triggered cancer therapy approach, with the overall goal to enhance the antitumor efficacy by the enhancement of drug accumulation in tumor region and reduced side-effects of PLGA-based DDSs.

Although a variety of inorganic or organic materials have been utilized to fabricate the smart DDSs including inorganic nanoparticles, liposomes, polymer micelles, and polymer nanoparticles which can be used to co-deliver therapeutic agents and imaging agents for cancer theranostic applications, several problems are still needed to be solved, such as the complicated synthesis process, non-biodegradability, and uncontrollable drug release behavior of some DDSs. For instance, the inorganic nanomaterials such as gold and silica which have poor biodegradability are most likely to cause the long-term toxicity and raise the safety concerns. And other metal nanomaterials such as copper and silver also exhibit some cytotoxicity. Moreover, the uncontrollable drug release behavior of some DDSs is highly likely to cause the compromised anticancer effect. However, polymer nanoparticles based on poly(D,L-lactic-co-glycolic acid) (PLGA) have attracted considerable attention due to their unique physical and chemical properties, such as tunable particle size, uniform and regular morphology, large surface area, favorable pharmacokinetic property, excellent biocompatibility and biodegradability. More importantly, PLGA has been approved for the medical applications by the United States FDA, making them a favorable material to construct the nano- or micro-platforms for medical applications. Unlike the polymer nanoparticles based on poly(butylcyanoacrylates) (PBCA) of which the hydrolysis products are cytotoxic polycyanoacrylic acid and alcohol, the PLGA-based polymer nanoparticles can be degraded into the non-toxic lactic acid and glycolic acid, which can be eventually degraded into water and carbon dioxide. The biocompatible and biodegradable nature of PLGA makes them preferentially used to prepare the nano- or micro-platform. And the appropriate outer surface engineering (such as PEGylation, BSA modification and conjugation of targeting ligands) of PLGA-based DDSs can endow them with a prolonged blood circulation and enhanced accumulation in tumor sites.

Besides the hydrophilic therapeutic drugs and imaging agents, the hydrophobic therapeutic drugs and imaging agents can be encapsulated into the PLGA nanoparticles by using the water-in-oil-in-water (W/O/W) double emulsion method, which can improve the targeting and bioavailability of hydrophobic drugs and imaging agents, and eventually achieving the goal of cancer theranostics. However, it is not appropriate to design the extremely complicated nano- or micro-platform based on PLGA, since their unstability or potential uncertainty in physiological environment may lead to the unfavorable tumor inhibition effect. How to design and build a versatile but simple drug delivery systems with the effective antitumor activity remains a challenge for the new research. Employing external stimuli to trigger the cancer therapy is a promising antitumor approach, which can selectively and precisely eradicate the solid tumors and remotely control the drug release. However, the applications of remotely triggered cancer therapy remains limited. For example, the PDT efficacy will be weakened with the oxygen consumption during PDT (Wang et al., 2017). Developing a strategy that can afford sufficient oxygen for photosensitizers to consume and continuously generate ROS, has been proved to be a promising approach for enhancing PDT efficacy (Tang et al., 2018). In addition, the tissue penetration depth of the irradiation light is an essential issue for the photo-triggered therapeutic application. Employing the light in the NIR region may address this issue due to their advantages of real-time dosage adjustment, high spatiotemporal precision, and deep tissue penetration (Lin et al., 2016; Zhang P. et al., 2016; Shen et al., 2019).

Although the PLGA-based DDSs have been extensively studied, which can provide a safe and reliable approach for the tumor diagnosis and therapy in a minimally invasive manner, most of them are just preclinical studies, and still face many challenges. The property of low drug loading, high production costs, and inability to mass-produce of PLGA-based DDSs may limit their clinical applications. In order to apply the PLGA-based DDSs load with diagnostic and therapeutic agents to the clinic, the researchers should firstly overcome the obstacles of large-scale production of the nanoparticles. And it will provide a solid foundation for the application of PLGA-based DDSs in tumor therapy if more methods are provided to improve the drug loading capacity of PLGA-based DDSs. In addition, the cytotoxicity and immune response of all components of the PLGA-based DDSs in the body should be evaluated. The clinical transformation of PLGA-based DDSs can be further promoted with the continuous development of materials science and nanotechnology. And the great efforts have been made to improve their biocompatibility, stability, safety, drug loading capacity, and targeted delivery, which will endow PLGA-based DDSs with great potential in cancer diagnosis and treatment.

## Author Contributions

All authors listed have made a substantial, direct and intellectual contribution to the work, and approved it for publication.

## Conflict of Interest

The authors declare that the research was conducted in the absence of any commercial or financial relationships that could be construed as a potential conflict of interest.
